# Assessment of biophysical properties of the first-in-class anti-cancer IgE antibody drug MOv18 IgE demonstrates monomeric purity and stability

**DOI:** 10.1080/19420862.2025.2512211

**Published:** 2025-05-28

**Authors:** Paul Considine, Panida Punnabhum, Callum G. Davidson, Georgina B. Armstrong, Michaela Kreiner, Heather J. Bax, Jitesh Chauhan, James Spicer, Debra H. Josephs, Sophia N. Karagiannis, Gavin Halbert, Zahra Rattray

**Affiliations:** a Strathclyde Institute of Pharmacy and Biomedical Sciences, University of Strathclyde, Glasgow, UK; b Drug Substance Development, GlaxoSmithKline, Stevenage, UK; c Cancer Research UK Formulation Unit, Strathclyde Institute of Pharmacy and Biomedical Sciences, University of Strathclyde, Glasgow, UK; d St. John’s Institute of Dermatology, School of Basic and Medical Biosciences & KHP Centre for Translational Medicine, Guy’s Hospital, King’s College London, London, UK; e School of Cancer and Pharmaceutical Sciences, King’s College London, London, UK; f Cancer Centre, Guy’s and St Thomas’ NHS Foundation Trust, London, UK; g Breast Cancer Now Research Unit, School of Cancer & Pharmaceutical Sciences, King’s College London, Innovation Hub, Guy’s Cancer Centre, London, UK

**Keywords:** Cancer immunotherapy, formulation, IgE, immunoglobulin, monoclonal antibody, particle size, protein aggregation, stability

## Abstract

Therapeutic monoclonal antibodies, which are almost exclusively IgG isotypes, show significant promise but are prone to poor solution stability, including aggregation and elevated solution viscosity at dose-relevant concentrations. Recombinant IgE antibodies are emerging cancer immunotherapies. The first-in-class MOv18 IgE, recognizing the cancer-associated antigen folate receptor-alpha (FRα), completed a Phase 1 clinical trial in patients with solid tumors, showing early signs of efficacy at a low dose. The inaugural process development and scaled manufacture of MOv18 IgE for clinical testing were undertaken with little baseline knowledge about the solution phase behavior of recombinant IgE at dose-relevant concentrations. We evaluated MOv18 IgE physical stability in response to environmental and formulation stresses encountered throughout shelf life. We analyzed changes in physical stability using multiple orthogonal analytical techniques, including particle tracking analysis, size exclusion chromatography, and multidetector flow field flow fractionation hyphenated with UV. We used dynamic and multiangle light scattering to profile aggregation status. Formulation at pH 6.5, selected for use in the Phase 1 trial, resulted in high monomeric purity and no submicron proteinaceous particulates. Formulation at pH 5.5 and 7.5 induced significant submicron and sub-visible particle formation. IgE formulation was resistant to aggregation in response to freeze–thaw stress, retaining high monomeric purity. Exposure to thermal stress at elevated temperatures resulted in loss of monomeric purity and aggregation. Agitation stress-induced submicron and subvisible aggregation, but monomeric purity was not significantly affected. MOv18 IgE retains monomeric purity in response to formulation and stress conditions, confirming stability. Our results offer crucial guidance for future IgE-based drug development.

## Introduction

Monoclonal antibody (mAb)-based immunotherapies represent a sizeable portfolio of cancer therapies, with >100 antibodies approved or under clinical and regulatory evaluation as oncology therapeutics. Immunotherapies in the clinic are largely based on the immunoglobulin (IgG) class. However, in recent years, the therapeutic potential of recombinant antibodies with Fc regions belonging to immunoglobulin E (IgE)- commonly associated with mediating allergic responses- has emerged as a new modality for targeting solid tumors.

The unique characteristics of IgE such as a high affinity for cognate Fc receptors (100–10,000-fold higher than IgG for FcγRs),^1^ result in longer tissue residency time (*i.e.*, 2 weeks versus 3 days for IgG), based on retention of the antibody on the immune cell surface in the absence of immune complex formation,^2^ engagement with immune cells in the tumor microenvironment,^1^ and potent effector functions.^3^ The therapeutic potential of IgE-based immunotherapies has been evaluated in numerous preclinical studies against several target antigens to-date, demonstrating the potential IgE-based therapies hold for treating solid tumors.^3,4^ IgE has shown potential therapeutic benefit as an alternative to IgGs for cancer immunotherapy, due to its ability to mediate the stimulation of monocytes and macrophages toward pro-inflammatory states and the degranulation of mast cells, upon cross-linking of FcεRI, which is constitutively expressed on monocyte, macrophage, and mast cell surfaces.^5^ Release of pro-inflammatory cytokines, chemokines and mediators such as tumor necrosis factor (TNF) leads to anti-tumor immune responses.^6^

The first-in-class IgE antibody candidate, MOv18 IgE, recognizing the cancer-associated antigen folate receptor-alpha (FRα), completed a Phase 1 clinical trial in patients with solid tumors and treatment showed early signs of efficacy at a low drug dose.^7^ A recent report demonstrated that through process development and scaled Good Manufacturing Practice (GMP) production, MOv18 IgE retained the glycosylation, biophysical, and functional characteristics of the research grade material.^8^ Comparable attributes between research grade and GMP material included expected pH, purity, concentration and early evidence of stability properties. However, the process development of MOv18 IgE was undertaken with little baseline knowledge about IgE solution phase behavior under real-world storage and handling conditions.

While substantial strides have been made in engineering mammalian expression systems for the bioproduction of IgE in preclinical and clinical indications,^1,9^ and the therapeutic advantages of IgE are well characterized, there are no reports of IgE formulation and physical stability characteristics. Therapeutic antibody formulations must possess sufficient physical and chemical stability in their intended formulation presentation throughout the product lifecycle, from manufacture to patient administration, as they are prone to a range of destabilizing effects from physical and chemical insults. Pathways for antibody destabilization include chemical alterations (e.g., oxidation, deamidation, and glycation), biotransformation, and physical instability (aggregation and fragmentation).^10^ Aggregation is the unfolding and self-association of native proteins into high molecular weight multimer species which occurs at different size ranges. Antibody aggregates can be broadly categorized as reversible, irreversible, soluble, or insoluble in nature.^11,12^

Antibody aggregation can occur during any stage of the product lifecycle in response to environmental stressors, such as exposure to elevated temperature, freeze–thaw (FT) cycling, agitation stress during shipment, exposure to extreme pH fluctuations, and light exposure. Therefore, the control of these formulation variables is critical during formulation design. Excipients (osmolytes, salts) are used to enhance protein surface hydration and prevent unfolding.^13^ Surfactants (e.g., polysorbate 20/80, poloaxamer 188) reduce protein aggregation in cell culture medium^14^ *via* preferential adsorption of the surfactant to a location on the protein which inhibits self-association, or by stabilization of the protein conformation from surfactant binding to the protein native-state.^15^ Surfactants also play a crucial role in protein stabilization by interacting with the air-liquid and liquid-solid interfaces, preventing destabilizing interactions that lead to aggregation.^16^ They are commonly used in biopharmaceutical formulations to reduce antibody adsorption to hydrophobic surfaces, such as glass vial walls and tubing, during manufacturing.^17^

When exposed to acidic conditions (pH 4.0), IgGs form reversible aggregates, oligomers, and β-sheet structures,^18^ and deamidation of glutamine occurs at basic pH (pH 9).^19^ Overall, these trends in stability dictate that IgG-based mAbs are formulated at a neutral-acidic pH.^20–22^ Maintaining the monomeric purity of mAbs and controlling for aggregation events and fragmentation through formulation design is critical to mitigate for reduced therapeutic efficacy and increase immunogenicity. Thus, regulatory authorities require rigorous characterization of mAb stability during product development.^23,24^

In this study, we provide further insights into the stability of MOv18 IgE, in response to formulation pH and environmental stress conditions using multiple orthogonal analytical techniques. The formulation stability of MOv18 IgE was tested to determine optimal formulation conditions. To achieve this, the impact of formulation pH and environmental stresses (thermal, FT, and agitation stress) on IgE physiochemical stability were assessed under formulation concentrations (*i.e.*, 1 mg/mL).

## Materials and methods

### Materials

An IgE antibody molecule was provided by the Cancer Research UK Formulation Unit formulated at 1 mg/mL in a 0.1 M sodium citrate, 30 g/L L-arginine, 50 g/L sucrose, and 0.02% polysorbate 20 in water for injection (pH 6.5 ± 0.1) and stored at 2–8°C.

Sodium Citrate Dihydrate was obtained from Thermo Fisher Scientific (Fisher Scientific, Inchinnan, Renfrew, UK). Sucrose was purchased from Merck (Sigma-Aldrich, Poole, Dorset, UK), A14730 L-arginine monohydrochloride 98% from Alfa Aesar (Thermo Fisher Scientific, Lancashire, UK), Tween® 20 was purchased from VWR Chemicals (Avantor, Lutterworth, Leicestershire, UK).

### Methods

#### Sample preparation for stressed conditions

##### Thermal stress protocol

**Figure 1.**
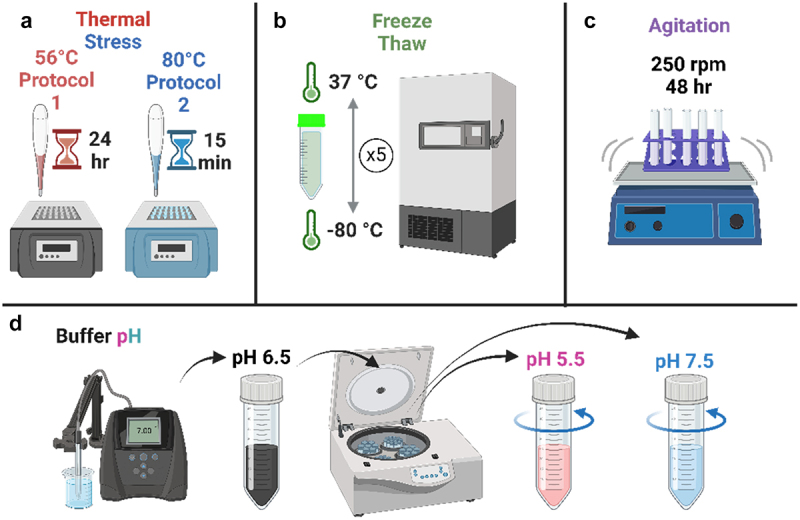
Sample preparation for mimicking stressed conditions.

IgE response to thermal stress was measured using two different thermal stress protocols. Aliquots (2 mL) of IgE, provided by the supplier at a final concentration of 1 mg/mL, were incubated in low protein-binding microcentrifuge tubes on a heat block at 56°C for 24 hours (protocol 1). This temperature was selected as the point at which the IgE Fc region begins to unfold, and aggregation is induced.^25^ To guarantee aggregation, IgE was heated separately to 80 °C for 15 min using the same experimental setup as protocol 1 (protocol 2), as most proteins reach Tm between 40 °C and 80 °C.^11^ All thermal stress samples were cooled to ambient temperature prior to the analysis of oligomer content and particle size (Figure 1a, Figure S1).

IgE at 1 mg/mL was analyzed from baseline untreated at pH 6.5, after exposure to 56 °C for 24 h or 80 °C for 15 min (a). After five freeze–thaw cycles (FT) (b). After 48 hours shaking (agitation stress) (c), or following buffer exchange into pH 5.5 and pH 7.5 (d). (Created with BioRender.com).

##### Freeze–Thaw stress

An uncontrolled FT protocol was used to replicate the effect of extreme temperature fluctuations on IgE aggregation, which may be encountered during the storage and transportation of antibody therapeutics. Filtered IgE was aliquoted into low protein binding microcentrifuge tubes placed on a sample rack and stored in a −80 °C freezer for 1 hour. The samples were thawed for 30 min at 37 °C in a constant temperature room to mimic exposure to high temperatures in warmer climates. This process was repeated five times before analysis (5 FT cycles) (Figure 1b).

##### Agitation stress

Agitation stress is a physical stress mAbs encounter during production (cell culture, purification, formulation, product filing) and shipping.^12^ The response of IgE to agitation stress was measured by 48-h incubation on a Stuart™ Microtitre Plate Shaker SSM5 (Cole-Parmer™, Cambridgeshire, United Kingdom) at 250 revolutions per minute (rpm) under refrigerated temperature (4 °C) conditions, in Eppendorf® Safe-Lock micro test tubes (Cat No. EP0030120230 Sigma-Aldrich, Poole, Dorset, UK), with a 2 mm headspace for each sample tube (Figure 1c).

##### Buffer pH

To evaluate IgE stability at different pH, the formulation was exchanged into citrate buffer (sodium citrate, 30 g/L L-arginine, 50 g/L sucrose, 0.02% TWEEN 20, pH 6.5- baseline), pH 7.4 and pH 5.5 using Amicon® Ultra-15 Centrifugal Filter units at 2600 × g (Merck Milipore, Dorset, Gillingham, UK). A microplate Bradford (Pierce^TM^ Coomassie Plus Assay Kit, Thermo Fisher, Loughborough, Leicestershire, UK) assay was performed to confirm protein sample concentration following buffer exchange. Absorbance of internal standards and buffer-exchanged samples was read at 600 nm using a GloMax® Explorer Multimode Microplate Reader (Promega Corporation, Chilworth, Southampton, UK) and the concentration determined from internal standard calibration plots (Figure 1d).

#### Analysis of baseline and stressed samples

##### Size exclusion chromatography (SEC)

A High-Performance Liquid Chromatography (HPLC) Shimadzu LCMS-2050 (High-Performance Liquid Chromatography Mass Spectrometer) (Shimadzu, Wolverton, Milton Keynes) with a TSKgel® SuperSW mAb HTP HPLC column (150 mm × 4.6 mm, 4 µm particle size, TOSOH Bioscience, Tokyo, Japan) was used for SEC analysis. Elution was performed using phosphate-buffered saline (PBS) pH 7.4 as the mobile phase at a flow rate of 0.35 mL/min. Peaks corresponding to IgE were detected at 280 nm with a photo diode array (PDA) detector. Samples were injected at a concentration of 1 mg/mL (10 µL injection volume). Quantification of different molecular weight species was performed by integrating the area under the peak following baseline subtraction. The areas corresponding to monomer and high molecular weight species were calculated from chromatogram peaks and expressed as a percentage of the total chromatogram peak area (%).

##### Asymmetric flow field flow fractionation AF4-UV-MALS

An AF2000 (PostNova Analytics, Worcestershire, UK) hyphenated with multiangle light scattering (MALS-PostNova Analytics, Worcestershire, UK) and UV detection (280 nm-PostNova Analytics, Worcestershire, UK) was used to analyze the monomeric purity of all samples at baseline and following stress (1 nm−500 microns). A channel spacer of 350 µm thickness and a 10 kDa molecular weight cutoff (MWCO) amphiphilic regenerated cellulose membrane were used for all measurements at ambient temperature (25°C), with a 100 µL injection loop size and a 20 µL injection volume. Elution conditions included an injection flow rate of 0.2 mL/min, a crossflow rate of 2 mL/min, and a detector flow rate of 0.5 mL/min at ambient temperature using 1 × PBS (pH 7.4) as the carrier liquid (Figure S2). System performance was verified with bovine serum albumin (BSA) and gamma-globulin standards. The percent void peak volume, resolution, and percent mass recovery were optimized for AF4 methodology. The AF4 methodology was developed in accordance with ISO/TS 21,362:2021, which specifies the standard parameters required for validating FFF-based methods. This standard includes a minimum sample recovery threshold of ≥ 70% and requires that the method of recovery be reported, using the following equation:

(1) $$\%\mathrm{Recovery}=\frac{\mathrm{Total}\;\mathrm{Mass}}{\mathrm{Injection}\;\mathrm{Mass}}\times 100$$

##### Dynamic light scattering (DLS)

The particle size distribution for IgE was measured by dynamic light scattering (DLS), using a Zetasizer Nano ZS90^TM^ (Malvern Panalytical, Malvern, Worcestershire, UK) using the 173° noninvasive backscatter setting (NIBS). The corresponding protein parameters selected were a refractive index of 1.45, and an absorption of 0.001 au corresponding to protein for the samples. All IgE samples were measured at 1 mg/mL and equilibrated at 25 °C prior to measurement. The number of runs for each replicate was optimized for each sample prior to the initiation of measurements. Corresponding Z-average, polydispersity index (PDI) and intensity-based size distribution were recorded on the Zetasizer Nano software v3.30^TM^ (Malvern Panalytical, Worcestershire, UK). The same equipment and software were used to determine the diffusion coefficient at different concentrations (0.1−1.5 mg/mL) for baseline and pH stress samples and to subsequently calculate the diffusion interaction parameter (K_D_), a measure of self-association, using the following equation:

(2) $$D=D_{0}\left(1+k_{D}c+...\right)$$

Where D is the diffusion coefficient, D_0_ is the maximal diffusion coefficient, and c the protein concentration.^26^

##### Electrophoretic light scattering

Electrophoretic light scattering was performed to measure changes in the ζ-potential of the IgE both following buffer exchange into pH 5, pH 5.5, and pH 7.5 formulation buffer and from baseline (pH 6.5). All zeta potential measurements were performed at 25 °C with 120 s equilibration time.

##### IgE charge predictions and homology modelling

To predict IgE isoelectric points, published heavy and light chain sequences of an anti-high molecular weight melanoma associated antigen (anti-HMW-MAA)^27^ were inputted in FASTA format into the sequence editor of the Molecular Operating Environment (MOE) software (version 2020.0901, Chemical Computing Group, Montreal, Canada). Sequences were annotated in IMGT numbering before modeling the fragment antigen-binding region (Fab) using the *Antibody modeller* tool in MOE, selecting Fab as the model type. Default settings were used with capping of C-termini with neutral residues and a gradient refinement limit of 1. A framework match to PDB 3NZH were 79.1% and 86.8% for light and heavy chains, respectively. Light chain complementary-determining region (CDR) matches were 100% to PDB ID 4GMS (mouse), 100% to PDB ID 6VRQ (mouse) and 100% to PDB ID 4R97 (mouse) for CDRL1, 2 and 3, respectively. Heavy chain CDR matches were 95.9% to PDB ID 3NZH (human), 83.5% to PDB ID 7TXZ (mouse) and 57.6% to PDB ID 7LKH (mouse) for CDRH1, 2 and 3, respectively. The final Fab model structure was corrected with the *Structure Preparation* function in MOE before energy minimization. A 0.1 kcal/mol/Å^2^ root mean square gradient was used with no periodicity nor constraints for this minimisation. To model the Fc region, a published IgE Fc model was used as template (PDB archive ID: 1IGE) and the *Homology modeller* tool in MOE was used with default settings (refinement gradient limit set to 0.5, Generalized Born/Volume Integral used for calculating electrostatic solvation energy). The top scoring Fc model (lowest RMSD to mean, contact energy and fewest outliers) was selected. This model had an additional 10-residues in the heavy chain prior to the CH2 domain, which was consistent with the template as well as an IgE crystal structure sequence (PDB: 5 MOL). The final Fc structure was corrected, and energy minimized similarly to the Fab model. The Fab model was then duplicated and joined to Fc model before final structure correction and protonation to pH 6 with the *Protonate 3D* function in MOE. Finally, the *Protein Properties* tool in MOE was used to compute sequence (pI_seq) and structure based (pI_3D) isoelectric points as well as predicted zeta potential at Deybe length, computed at an arbitrary ionic strength of 0.1 M NaCl. The variable region (fV) was also extracted from this model, partial charges corrected and protonated to pH 6 as a separate model to distinguish charge contributions from Fc versus Fv.

##### Nanoparticle tracking analysis (NTA)

Nanoparticle tracking analysis (NTA) was used to measure changes in IgE particle size and concentration in response to different stresses at 1 mg/mL, with a focus on particulates in the submicron/subvisible size range (10–2000 nm). A Nanosight NS300^TM^ configured with a 488 nm laser and a high sensitivity CMOS camera (Malvern Panalytical, Malvern, Worcestershire, UK) was used to measure all samples under a constant flow rate (100) through a syringe driver at ambient temperature (25°C), with five, 60-s videos acquired at a camera level of 5 (thermal stress) or 10 (FT, pH, and agitation stress).

The corresponding particle size distribution span was determined using the following equation:

(3) $$\mathrm{Span}=\frac{D_{90-}D_{10}}{D_{50}}$$

All data were processed in the NS300 NTA software v3.4^TM^ with a detection threshold of five used for all experiments.

##### Gel electrophoresis

Sodium dodecyl-sulfate polyacrylamide gel electrophoresis (SDS-PAGE) was performed using 4–20% Mini-PROTEAN^TM^ TGX Precast Gels (Bio-Rad Laboratories, Watford, Hertfordshire, UK) and Tris-Glycine-SDS Running Buffer (Cell Signalling Technology, London, UK) diluted in Milli-Q® water (Merck Millipore, Watford, UK) incubated in the Mini-PROTEAN^TM^ Tetra Cell (Bio-Rad Laboratories, Watford, Hertfordshire, UK). Prior to loading onto the gel lanes, samples were mixed with sample buffer and heated to 92°C. Precision Plus Protein^TM^ Standards Kaleidoscope^TM^ (Bio-Rad Laboratories, Watford, Hertfordshire, UK) was used as the molecular weight marker. The electrode voltage was 250 V and the gel ran for 30–35 min. All gels were stained with a QC Colloidal Coomassie Stain (Bio-Rad Laboratories, Watford, Hertfordshire, UK) using the rapid stain protocol as per manufacturer instructions.

##### Hydrophobic interaction chromatography (HIC)

A TSKgel® Butyl-NPR HPLC HIC Column (TOSOH Biosciences, Griesheim, Germany) was installed in a ACQUITY Premier UPLC system (Waters, Massachusetts, USA) for assessing net hydrophobicity of IgE samples in PBS with 10 μg injections. A mobile phase consisting of 1.5 M ammonium sulfate in phosphate buffer was used for a 20-min cycle time with a 0–100% gradient to phosphate buffer with organic modifier. Empower (version 3.8.1, Waters, Massachusetts, USA) was used for extracting integrated chromatograms and raw data that was then processed (baseline and blank subtracted and averaged) offline in an in-house Python script (version 3.8.20) and executed in a Jupyter Notebook environment (version 7.2.2).

### Data and statistical analysis

All data were reported as mean (± standard deviation). Unless otherwise stated, all variables were measured with three replicates in each measurement, and three independent replicates for each measurement. A Tukey simultaneous test for difference of means was performed using Minitab Statistical Software Version 21.1.0 (Minitab, State College, Pennsylvania) to assess the significant differences in monomer, fragment, and oligomer/aggregate percentage (%), NTA spans (nm), Z-average (nm), and PDI between the different stress conditions, where a p-value <0.05 was deemed as being statistically significant. Graph analysis was completed using Origin 2022 Build No. 9.900225 (OriginLab, Northampton, Massachusetts, USA).

To directly compare the oligomerization and aggregation states of IgE, we quantified the relative content (%) of IgE fragments, monomers, dimers, potential oligomers, and high-molecular-weight aggregates related to the UV signal for both AF4 and SEC techniques. During AF4, analytes eluting at 8–20 min corresponded to IgE monomers, dimers, and possible oligomers (molar mass 1.8 × 10^5^ − 9.5 × 10^5^ Da).^28^ Species eluting at 20–30 min were classified as “aggregates” (molar mass > 9.5 × 10^5^ Da). Analytes eluting between 6–8 min were classified as IgE fragments, since they eluted earlier than the monomer (Table S 1). Subsequent peak retention time data produced by SEC assumed that the most prominent peak in the pH 6.5, pH 5.5, pH 7.5, FT, and agitation samples were monomer (3.6–3.7 min), and the prominent peaks from 56°C to 80°C samples were aggregates (2.9–3 min) (Table S 2).

## Results

### IgE physical stability is dependent on formulation buffer pH

There are limited data available on IgE behavior under different pH conditions, and optimal pH is an important formulation parameter to modulate antibody solvent accessible charged patch coverage, and to control for protein–protein interactions.^29^ Additionally, evaluation of therapeutic mAb stability at low pH is an important condition to evaluate, as downstream mAb purification processes, such as protein A chromatography involve exposure to acidic conditions. Simultaneously, therapeutic mAb behavior at high pH must be evaluated because high pH elution buffers are used during anion exchange chromatography.^12^ To simulate different pH conditions and confirm their impact on IgE stability, samples were buffer exchanged into citrate buffer (sodium citrate, 30 g/L L-arginine, 50 g/L sucrose, 0.02% Tween 20) from pH 6.5 (baseline, 1 mg/ml) to pH 7.4 and pH 5.5 (both 1 mg/ml) (Figure 1 1d), and analyzed immediately from refrigerated conditions (2–8°C).

Visual assessment was performed immediately following buffer exchange to rule out the presence of particulates, and all test solutions were confirmed as clear and not opalescent in appearance.

**Figure 2.**
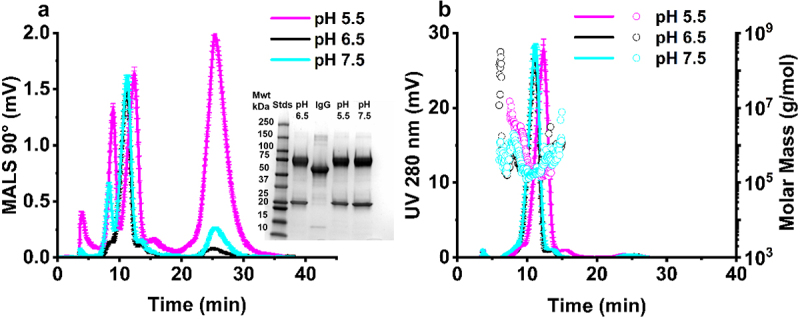
Characterization of pH effects on IgE monomeric purity. Corresponding AF4-MALS 90° fractogram (a), AF4-UV fractogram trace (y-axis left) measured at 280 nm, and corresponding AF4-MALS molar mass distribution (g/mol) (y-axis right) (b), SDS-PAGE analysis of molecular weight (a) inset. All samples were formulated at 1 mg/mL. Error bars represent ± standard deviation (*N* = 3) (2–8 °C). mwt – molecular weight, STDs – weight standards, BL – baseline, IgG – IgG standard.

**Figure 3.**
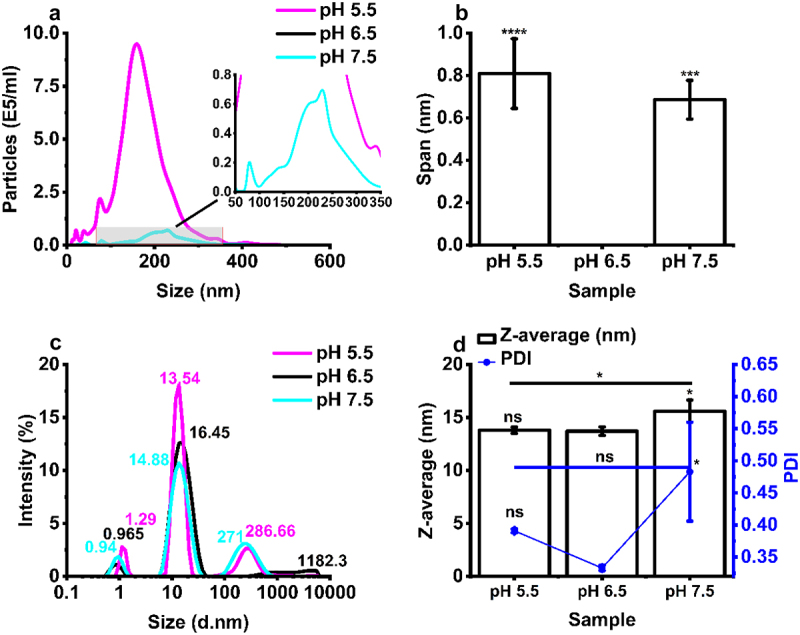
Formulation of IgE at different formulation buffer pH leads to the formation of submicron particles. (a) Analysis of size distribution by nanoparticle tracking analysis (NTA) (b) particle size span determined by NTA, (c) analysis of size distribution by dynamic light scattering (DLS) intensity-based size distribution, and (d) Z-average and polydispersity index (PDI) determined by DLS. All IgE samples were analyzed at 1 mg/mL in pH 5.5, 6.5 and 7.5 buffer (2–8 °C). Error bars represent mean ± standard deviation (*N* = 3), statistical analysis was completed using a tukey test *p < 0.05, **p < 0.01, ***p = 0.001, ****p < 0.001, ns – not significant versus pH 6.5 unless indicated.

Exposure to formulation conditions in the pH 5.5–7.5 range led to changes in the AF4-MALS 90° fractogram (Figure 2a), accompanied by a later eluting peak for both pH 5.5 and pH 7.5 samples, measured by AF4-UV at 280 nm for pH 5.5 samples, under refrigerated conditions for 24 hours (2–8 °C) (Figure 2b). This observed shift in elution time was accompanied by significant sub-population peaks seen in the AF4-MALS trace, observed in the case of pH 5.5 and pH 7.5 samples (Figure 3a). There were no statistically significant changes observed in molar mass (g/mol) (Figure 2b) or monomeric purity (Table 1) or fragment molecular weight (Figure 2a inset) for the pH conditions test. These results were validated by SEC-UV, where minimal changes in retention time (Figure S3) and purity (Table 1) were observed as with the AF4-UV-MALS data.

**Table 1.** Percentage monomeric purity calculated from AF4-UV-MALS and SEC-UV pH data.

AF4-UV-MALS	Recovery % (±SD)	Fragment % (±SD)	Monomer % (±SD)	Oligomer % (±SD)	Aggregate % (±SD)
pH 5.5	101.35 (±0.78)	3.09 (±0.70) (**)	94.25 (±1.18)(ns)	2.95 (±0.41)(ns)	0.17 (±0.09) (****)
pH 6.5	85.41 (±1.72)	1.15 (±0.05)	93.62 (±1.01)	2.61 (±0.29)	3.17 (±0.33)
pH 7.5	97.71 (±1.30)	3.44 (±0.12) (**) [ns]	91.54 (±0.49) (ns) [*]	2.37 (±0.24)(ns) [ns]	2.37 (±0.17) (*) [****]
SEC-UV	Fragment % (±SD)		Monomer % (±SD)		Aggregate % (±SD)
pH 5.5	10.42 (±0.50) (ns)		87.66 (±0.92) (ns)		1.92 (±1.22) (ns)
pH 6.5	4.08 (±4.24)		93.72 (±4.99)		2.20 (±1.23)
pH 7.5	1.2 (±0.15) (ns) [ns]		94.17 (±1.13) (ns) [ns]		4.63 (±0.98) (ns) [ns]

IgE (1 mg/mL) from untreated samples (pH 6.5) and buffer exchanged samples (pH 5.5 and 7.5) (2–8°C) (*N* = 3). Monomeric purity is referred to as monomer %. Data represent mean ± standard deviation, statistical analysis was completed using a tukey test*p < 0.05, **p < 0.01, ****p < 0.001, ns – not significant, () – versus pH 6.5, [] – versus pH 5.5.

The size distribution of IgE across the pH range of 5.5 to 7.5 showed a notable increase in the radius of gyration (R_g_), as determined by AF4-MALS analysis. The R_g_ values for monomeric species remained relatively consistent within the range of ~7–11 nm across all pH conditions, while oligomeric species displayed larger R_g_ values, ranging from ~16 to 37 nm. The aggregate populations at pH 5.5 (~17 nm R_g_) and pH 7.5 (~76 nm R_g_) differed from those observed at pH 6.5 (~25 nm R_g_), indicating variations in aggregation behavior under different pH conditions (Table S 3).

Formulation of IgE at pH 5.5 and pH 7.5 resulted in the occurrence of submicron particles as detected by DLS intensity-based size distribution (peaks occurring at 286 and 271 nm), respectively (Figure 3c). No sub-micron particulates were detected by NTA for the pH 6.5 formulation samples, with a significant number of particles detected by NTA in the 10–1,000 nm size range, with 1.02 × 10^8^ (±3.18 × 10^7^) and 7.92 × 10^6^ (±1.80 × 10^6^) particles/mL for pH 5.5 and pH 7.5 samples, respectively, confirming the presence of sub-micron aggregates in these formulations (Figure 3a, Table S4). Formulation at pH 5.5 and 7.5 also resulted in a larger NTA particle size span compared to baseline samples, showing the presence of multiple particle size subspecies in the submicron size range for the pH exchanged samples (Figure 3b). Interestingly, formulation at pH 7.5 resulted in an increase in IgE Z-average and polydispersity index (PDI), as measured by DLS (0.3 nm−10 μm), relative to baseline (Figure 3d), while formulation at pH 5.5 did not result in a change in these parameters (Figure 3d).

Analysis of the diffusion self-interaction parameter calculated from the diffusion coefficient of the IgE as a function of sample concentration, resulted in a K_D_ parameter of −0.095 mL/mg in pH 6.5 formulations, 2.185 mL/mg for pH 5.5 samples, and 2.297 mL/mg for pH 7.5 IgE samples. The K_D_ self-interaction parameter was found to not be predictive of the colloidal stability of IgE (Figure S 4). Overall, our results obtained from the analysis of IgE monomeric purity and sub-micron particulate formation in response to formulation pH, confirms high percent monomeric purity (>90%) and the absence of submicron aggregates at pH 6.5. Therefore, the ability of IgE formulated at pH 6.5 to retain its stability in response to environmental stressors was explored further.

### IgE zeta potential measurements

**Figure 4.**
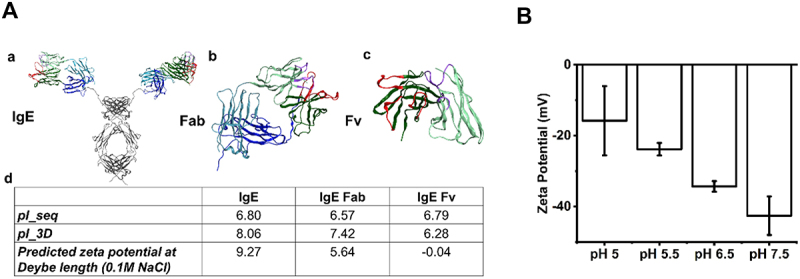
*In silico* prediction and experimental confirmation of IgE isoelectric point (pI). Homology modelling predicts charge parameters for IgE structure and sequence and structure-based isoelectric point. (a) Homology model to predict pI, conducted from the structure of anti-high molecular weight melanoma associated antigen IgE. For all structures, the CDRL 1, 2 and 3 (purple), CDRH 1, 2 and 3 (red), variable light chain region (light green), variable heavy chain region (dark green), light chain constant (light blue), heavy chain constant (dark blue) and Fc (grey) were annotated with IMGT numbering. The full IgE model (a), Fab region (b), and Fv region (c) models are shown with their respective sequence-based (pI_seq) and structure-based (pI_3D) isoelectric points as well as the zeta potential at pH 6, 0.1 M ionic strength (d). (b) Zeta potential measurements for all IgE samples were performed at 1mg/mL in pH 5, pH 5.5, 6.5 and 7.5 buffer (2–8 °C), to confirm that decreasing pH leads to IgE surface neutrality. Error bars represent ± standard deviation (*n*=3).

Homology modeling was used to predict the sequence and structure-based isoelectric point of IgE (Figure 4a), with these values ranging between 6.80 (sequence-based predicted isoelectric point (pI)) and 8.06 (3D isoelectric point). Analysis of the IgE zeta potential in formulation conditions was performed at different pH (Figure 4b) to assess the impact on the intact molecule’s behavior in solution. This indicates an isoelectric point occurring below pH 5.0. Zeta potential was selected for low sample volume and time-efficient screening.

### Thermal stress leads to loss of IgE monomeric purity while IgE is resistant to freeze–thaw cycling

Temperature fluctuations including thermal stress and FT are commonly encountered during temperature excursions resulting from transportation, storage, and handling of antibody-based drug products. To simulate the impact of temperature excursions and model irreversible aggregation, IgE samples (1 mg/mL) were subjected to thermal stress at 56 °C overnight (protocol 1), 80 °C for 15 min (protocol 2), or five FT cycles (−80 °C to 25 °C to −80 °C) (Figure 1a,b) before being left to cool to room temperature. Initial visual assessment of IgE samples following exposure to thermal stress and FT protocols, ruled out the presence of visible particulates and the formulation was clear in presentation.

While therapeutic antibodies are unlikely to encounter the selected thermal stress temperatures (56 °C and 80 °C), Arrhenius kinetic modeling has been used to predict long-term stability of antibodies at room temperature excursions (e.g., 20–37 °C) and long-term storage (2–8 °C), based on accelerated stability studies at elevated temperatures.^30^ The Tm-onset of the IgE Fc region (~56 °C) represents the beginning of unfolding, which can lead to aggregation and loss of function,^25^ while 80 °C induces irreversible denaturation, providing insights into worst-case degradation scenarios.^11^ These approaches allow manufacturers to estimate shelf life and assess the impact of brief temperature deviations during transport, ensuring robust stability predictions for commercial development.^31^

**Figure 5.**
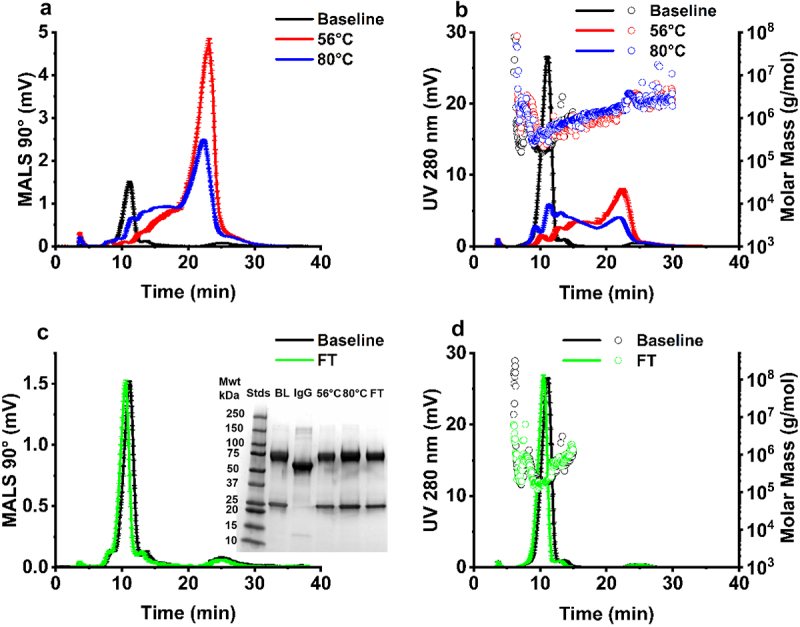
The effect of thermal stress and freeze-thaw protocols on IgE physical stability. AF4-MALS 90° fractogram trace (a) and AF4-UV at 280 nm fractogram trace (y-axis left) and corresponding AF4-MALS molar mass distribution (g/mol) (y-axis right) (b) for samples untreated (baseline) and exposed to 56 °C for 24 hours and 80 °C for 15 min. Corresponding AF4-MALS trace in response to freeze-thaw (5×) (c) and AF4-UV trace (y-axis left) and AF4-MALS molar mass distribution (g/mol) (y-axis right) (d), SDS-PAGE analysis of molecular weight (c, inset). All samples were formulated at 1 mg/mL and pH 6.5. Error bars represent mean ± standard deviation (*N* = 3). mwt – molecular weight, STDs – weight standards, BL – baseline, IgG – IgG standard.

Exposure to 56 °C and 80 °C thermal stress protocols led to a significant loss of IgE monomeric purity as analyzed by AF4-UV-MALS and SEC-UV (Table 2), accompanied by a significant increase in the occurrence of higher molecular weight species detected in comparison to IgE samples stored under refrigerated conditions (2–8°C), and significant shifts in elution time (Figure 5a,b). These data were confirmed by SEC-UV analysis, where aggregated peaks were observed (Table 2). Monomeric purity was not significantly affected by FT stress (Figure 5c,d, Table 2). Analysis by SDS-PAGE did not show any significant changes among different stress conditions (Figure 5c inset).

**Table 2.** Percentage monomeric purity calculated from AF4-UV-MALS and SEC-UV thermal stress and freeze–thaw data.

AF4-UV-MALS	Recovery % (±SD)	Fragment % (±SD)	Monomer % (±SD)	Dimer % (±SD)	Oligomer % (±SD)	Aggregate % (±SD)
Baseline	85.41 (±1.41)	1.15 (±0.05)	93.62 (±1.01)	nd	2.61 (±0.29)	3.17 (±0.33)
56°C	93.46 (±0.39)	0.76 (±0.03) (****)	4.11 (±0.09) (****)	8.61 (±0.28) (****)	25.95 (±2.86) (****)	60.57 (±3.03) (****)
80°C	91.91 (±0.68)	0.41 (±0.01) (****)	6.56 (±0.09) (****)	17.37 (±0.38) (****)	41.22 (±0.14) (****)	34.44 (±0.43) (****)
FT	77.57 (±0.99)	1.92 (±0.10) (***)	92.55 (±0.25) (ns)	nd	2.73 (±0.11) (ns)	2.80 (±0.16) (ns)
SEC-UV	Fragment (%)		Monomer (%)		Aggregate (%)	
Baseline	4.08 (±4.24)		93.72 (±4.99)		2.20 (±1.23)	
56°C	1.28 (±0.6) (ns)		nd		98.72 (±0.60)	
80°C	1.61 (±1.35) (ns)		nd		98.39 (±1.35)	
FT	1.84 (±0.95) (ns)		96.93 (±1.09) (ns)		1.23 (±0.14) (ns)	

IgE (1 mg/mL) from samples untreated (pH 6.5) (2–8 °C), exposed to 56 °C for 24 hours and 80 °C for 15 min, and 5 × freeze–thaw cycles (FT) (*N* = 3). Monomeric purity is referred to as monomer %. Data represent mean ± standard deviation, statistical analysis was completed using a Tukey test ***p = 0.001, ****p < 0.001, ns – not significant versus baseline, nd – not detected.

The size distribution profiled under thermal stress conditions (56 and 80°C) showed an alteration in the radius of gyration (R_g_), with AF4-MALS analysis (Table S 3) relative to the baseline. Dimer IgE species were observed following thermal stress, with R_g_ values of ~14 nm at 56 °C and ~32 nm at 80 °C, whereas no such species was detected under baseline conditions. The R_g_ values for monomeric species remained relatively consistent within the range of ~7–16 nm across all thermal stress conditions, while oligomeric species displayed larger R_g_ values, ranging from ~13 to 16 nm (Table S 3). However, no significant changes in R_g_ were observed in the aggregate populations at 56 °C (~24 nm) and 80 °C (~25 nm) relative to the baseline (~25 nm) (Table S 3). In addition, the R_g_ values of the aggregates increased to approximately 41 nm under FT stress conditions, compared to the baseline (Table S3). However, significant standard deviations were observed across the IgE size distribution data, indicating considerable variability.

**Figure 6.**
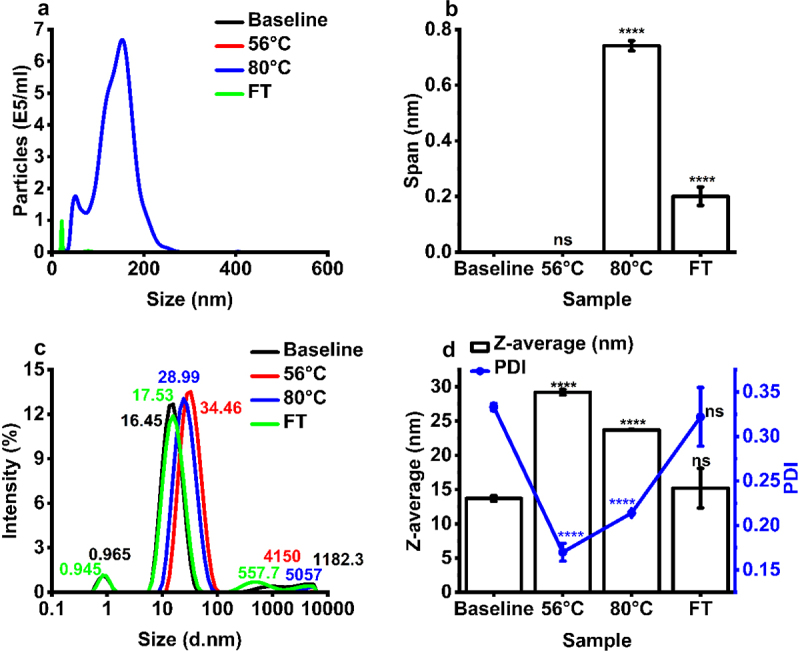
The effect of thermal and freeze-thaw stress on IgE aggregation status. Analysis of IgE size distribution by (a) nanoparticle tracking analysis (NTA), (b) NTA span, (c) dynamic light scattering (DLS) intensity-based size distribution used to calculate, and (d) Z-average and polydispersity index (PDI) determined from DLS. Mean ± standard deviation (*N* = 3). Statistical analysis was performed using a Tukey simultaneous test for difference of means - ****p < 0.001, ns – not significant versus baseline.

We next measured the exposure of IgE to thermal stress at 56 °C and 80°C (Figure 5). No particles were detected by NTA for control IgE samples (pH 6.5, 4 °C) and for samples stressed at 56 °C (thermal protocol 1), implying the absence of larger sub-micron aggregates (Figure 6a). NTA measured the presence of negligible submicron particles in samples exposed to FT stress (3.83 × 10^5^ ±2.72 × 10^5^ particles/mL), and a significant increase in submicron particles following thermal stress at 80 °C (thermal protocol 2) (5.85 × 10^7^ ±1.33 × 10^6^ particles/mL). These findings imply that aggregates formed at 80 °C are mostly large submicron particulates (Figure 6a, Table S4). Thermal stress at 80 °C resulted in a larger NTA particle size span compared to FT samples, showing the presence of multiple particle size subspecies in the submicron size range for the samples subjected to higher temperature stress conditions (thermal stress protocol 2) (Figure 6b). Thermal stress at 56 °C and 80 °C resulted in an increase in IgE particle size as measured by DLS (0.3 nm−10 μm), relative to baseline (Figure 6c,d), while FT did not significantly alter IgE particle size (Figure 6c,d). Moreover, a retention time shift and peak broadening was observed with thermally stressed IgE in hydrophobic interacting chromatography, indicating increased net hydrophobicity and increased polydiversity in hydrophobic species (Figure S5). No change in HIC profile was observed for the FT samples.

### IgE shows stability in response to agitation stress

Agitation stress is another physical stress that mAbs encounter throughout the mAb product lifecycle, which can occur during cell culture, purification, formulation, product filling, and shipping. Agitation stress increases the likelihood of mAbs encountering hydrophobic interactions that occur at the air-liquid interfaces.^12^ To simulate the impact of agitation stress resulting from shaking, IgE samples (1 mg/mL) were subjected to a 48 hour incubation on a plate shaker at 250 rpm under refrigerated conditions (2–8 °C), before analysis.

Visual assessment of the samples subjected to agitation stress was carried out immediately following agitation, confirming the absence of turbidity, opalescence, and visible particulates. Next, control and agitated samples were analyzed for changes in monomeric purity and sub-micron particulate formation.

**Figure 7.**
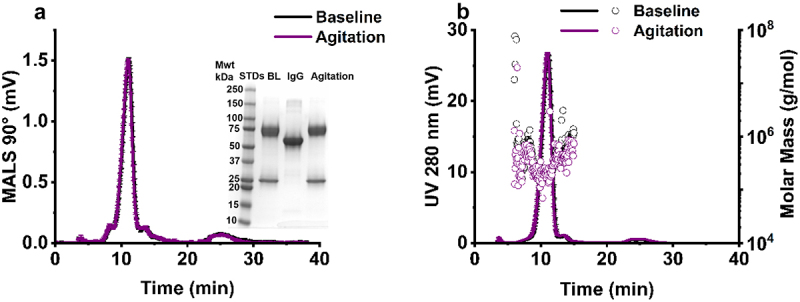
AF4-UV-MALS analysis of IgE following agitation stress. Agitation minimally changed elution time (a-b), molar mass (g/mol) (b), and molecular weight (a inset). IgE (1 mg/mL) from baseline untreated (pH 6.5) and after 48 hours shaking agitation stress at 250 rpm (2–8 °C), mean ± standard deviation (*N* = 3). mwt – molecular weight, STDs – weight standards, BL – baseline, IgG – IgG standard.

Exposure of IgE to agitation stress for 48 hours did not significantly change monomeric purity (Table 3), or the fragment molecular weight (Figure 7a inset) compared to baseline samples stored under refrigerated conditions (2–8 °C) conditions in the absence of agitation (Figure 7). No changes in the elution time for AF4-UV-MALS profile (Figure 7b) nor the occurrence of high molecular weight species (Figure 7b) were observed. This was confirmed by SEC with inline UV, where minimal changes in monomer retention time (Figure S3) and purity were observed (Table 3).

**Table 3.** Percentage monomeric purity calculated in response to agitation stress from AF4-UV-MALS and SEC-UV.

AF4-UV-MALS	Recovery % (±SD)	Fragment % (±SD)	Monomer % (±SD)	Oligomer % (±SD)	Aggregate % (±SD)
Baseline	85.41 (±1.7241)	1.11 (±0.05)	93.62 (±1.01)	2.61 (±0.29)	3.17 (±0.33)
Agitation	89.92 (±0.64)	1.64 (±0.04) (****)	92.13 (±1.99) (*)	3.20 (±0.13) (*)	3.03 (±2.14) (ns)
SEC-UV	Fragment % (±SD)		Monomer % (±SD)		Aggregate % (±SD)
Baseline	4.08 (±4.24)		93.72 (±4.99)		2.20 (±1.23)
Agitation	0.58 (±0.1) (ns)		98.36 (±0.14) (ns)		1.06 (±0.12) (ns)

IgE (1 mg/mL) from baseline control untreated samples (pH 6.5) and after 48 hours shaking agitation stress at 250 rpm (2–8°C) (*N* = 3). Monomeric purity is referred to as monomer %. Data represent mean ± standard deviation, statistical analysis was completed using a Tukey test *p < 0.05, ****p < 0.001, ns – not significant versus baseline.

The size distribution following exposure to 48 hours of agitation stress resulted in changes in the radius of gyration (R_g_), as determined by AF4-MALS. The R_g_ values for monomeric species remained relatively consistent, within the range ~7–15 nm, under both conditions, while oligomeric species displayed larger R_g_ values, ranging from ~11 to 16 nm. Despite these changes, the aggregate populations under agitation stress (~31 nm R_g_) did not differ significantly from the baseline values (~25 nm for R_g_) (Table S 3). Additionally, substantial standard deviations in IgE size distribution were observed across different formulations, indicating variability and inconsistency.

**Figure 8.**
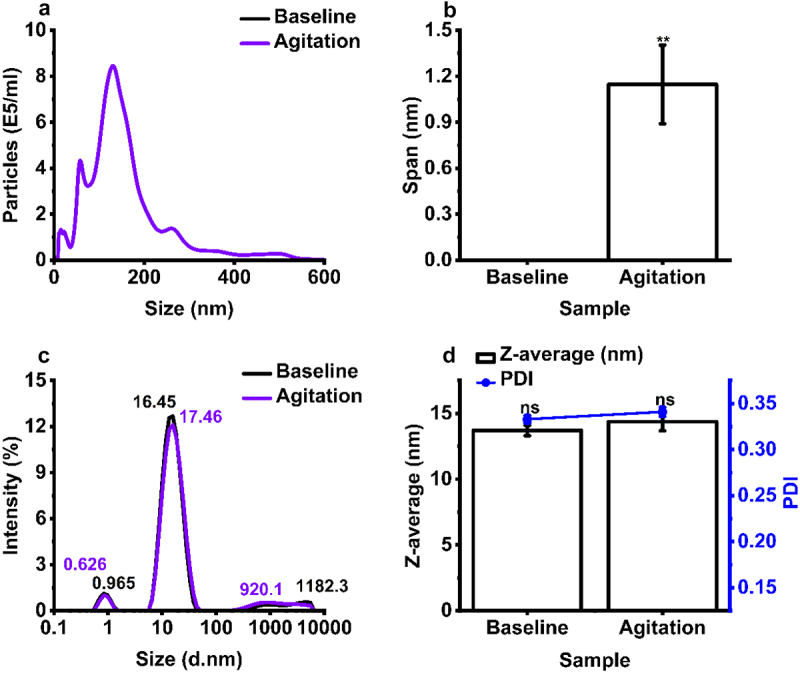
Agitation stress induces the formation of submicron size aggregates. Analysis of IgE size distribution by (a) nanoparticle tracking analysis (NTA) and (c) dynamic light scattering (DLS) used to calculate, (b) NTA span, and (d) Z-average and polydispersity index (PDI). IgE (1 mg/mL) from baseline untreated and 48 hours agitation stress at 250 rpm (2–8 °C). Mean ± S.D. (*N* = 3), statistical analysis was completed using a Tukey simultaneous test for difference of means - **p < 0.01, ns – not significant versus baseline.

A significant number of particles (1.03 × 10^8^ ±1.85 × 10^7^ particles/mL) (Table S4) were detected by NTA (dynamic range: 10–1,000 nm), for agitated samples, confirming the presence of larger sub-micron amorphous aggregates (Figure 8a). Agitated IgE samples also exhibited a larger NTA particle size span in comparison to baseline, implying the presence of multiple aggregate subpopulations occurring in the submicron size range for samples subjected to agitation (Figure 8b). Agitation stress did not impact particle size measured by DLS, relative to baseline conditions (Figure 8c,d). However, a peak was observed at ~920 nm, corresponding to the formation of large submicron particles.

## Discussion

Mitigating for self-association and drivers of mAb aggregation remains a critical aspect of therapeutic development, which is known to subsequently impact therapeutic mAb efficacy, immunogenicity, and the occurrence of adverse events in patients.^32–34^ It is well recognized that the occurrence of aggregates resulting from mAb self-association result in elevated serum titers of antibody-neutralizing antibodies.^35^ The United States Pharmacopeia (USP) specifies limits on the size and concentration of micron-sized particles in protein drug formulations, to minimize the potential for adverse events resulting from mAb aggregation in patients.^36^ However, proteinaceous aggregates occurring in the submicron and subvisible range have also been implicated in rapid drug clearance,^37^ reduced function,^38^ and immunogenic reactions in recent years.^39^ Particles in the submicron range are better suited for cellular uptake and Fcγ receptor activation than aggregates in the micron range.^40^ Therefore, the US Food and Drug Administration and USP now recommend evaluating submicron (2–10 µm) and subvisible particulates (1–100 µm) during mAb formulation development.^41^

Our goal in this study was to provide new insights into the aggregation properties of a novel recombinant IgE, the first-in-class therapeutic candidate MOv18 IgE, under different pH conditions (pH 7.5 and 5.5), and in response to different stresses (thermal, FT, agitation) encountered during the product lifecycle using orthogonal analytical techniques. While the biological advantages of IgE from a therapeutic perspective are well characterized,^42,43^ the aggregation propensity risks of IgE therapies resulting from environmental and formulation stressors at dose-relevant concentrations are not reported in the literature. Therefore, for the first time, we developed a pipeline of orthogonal analyses to measure products of degradation, aggregates and fragments, to provide insights into the stability of an IgE formulation spanning the nanometer-submicron size ranges. While the techniques detailed here have been previously used to characterize IgG-based mAbs for submicron-subvisible particle formation,^44–47^ our data demonstrate that these techniques can also be applied to the evaluation of IgE formulations.

We assessed the impact of physical and formulation stressors on IgE monomeric purity by AF4 multiplexed with UV and MALS to derive information on sample concentration, particle size, and molar mass. AF4 provides a gentle separation approach, which is ideal for separating fragile molecules (reversible aggregates) in polydisperse samples. The simultaneous inline separation and analysis of different subspecies formed in response to formulation stress offers a higher resolution approach for analyzing aggregate formation in comparison to DLS and NTA. Compared to SEC, which is routinely used for the analysis of mAb monomeric purity, analytes experience minimal shear stress during AF4-based separation, and a broader analytical size range can be measured (1 nm to 10 µm).^48^ With inherently less shear stress experienced during AF4 injection, focussing and separation steps, higher antibody-channel and antibody -membrane interactions are expected, thus lowering overall recovery compared to SEC. Accounting for this, our AF4 methodology was optimized to meet current International Organisation for Standardisation guidelines citing recovery > 70% and high-performance separation recovery >90%.^49^ Interestingly, the DLS self-interaction parameter derived in this study did not provide an indication of IgE colloid stability.

The formulation stability of mAbs is pH-dependent, resulting from the ionization status of the IgE and charge distribution profile of solvent accessible patches, factors vital to maintaining folding and retention of secondary/higher order structure and preventing aggregation.^50^ The formulation pH of protein therapeutics is carefully considered and IgGs are often formulated at a neutral-acidic pH (pH 6–7).^20–22^ IgE samples formulated in pH 6.5 formulation buffer had a high monomeric purity (Figure 2 and Table 1) and a low concentration (0 particles/ml) of submicron sized (10–2,000 nm) aggregates (Table S4). The subvisible (10–100,000 nm) range was not explored further, as no condition indicated the presence of particles larger than one micron in size (Figures 3a, 6a and 8a). While no condition indicated the presence of particles above 1 µm, techniques which measure the subvisible particle (SVP) range, light obscuration (LO) and flow imaging microscopy (FI), will likely be required for future MOv18 IgE characterization. LO is the current standard pharmacopoeial technique for SVP analysis in the quality control of therapeutic proteins.^51,52^ The USP chapters < 787> and < 788> state that for therapeutic protein products for infusion or injection in containers of ≤100 mL, the average number of particles present per container should be <6,000 particles ≥10 µm and 600 particles ≥25 µm.^53,54^ Baseline MOv18 IgE formulated at pH 6.5 baseline falls within these guidelines. Following buffer exchange to pH 7.5 and pH 5.5, significant shifts of particle size to higher molecular weight species was observed as measured by AF4-MALS (Figure 2a), and significant submicron particle (10^6^−10^8^ particles/mL) formation was observed (Figure 3b,c, Table S4), compared to baseline (pH 6.5).

Zeta potential analysis revealed that the IgE surface charge was trending toward neutral at below pH 5 (Figure 4b). This implies that the isoelectric point (pI) of the IgE was below pH 5 and therefore aggregation was more severe from pH 5.5 buffer exchange samples (submicron particle concentration of 1.02 × 10^8^ ±3.18 × 10^7^) compared to pH 7.5 (7.92 × 10^6^ ±1.80 × 10^6^) and pH 6.5 (0) samples (Table S 4). Closer to the pI, a decreased charge of molecules diminishes the electrostatic repulsion between proteins and increases aggregation^55^ while a pH far away from the pI may also induce instability.^56^ Whilst the formulation composition was intended to be consistent across the three pH levels explored, it is important to note that the polysorbate 20 (PS20) concentration was not verified in this initial early screening experiment. Method development for PS quantitation is ongoing and serves as a crucial quality control measure for monitoring the oxidative degradation of these nonionic stabilizing surfactants.^57–59^

We next evaluated the response of IgE to thermal stress at elevated temperatures (56°C and 80°C), known to induce unfolding and aggregation of mAbs, respectively.^25,60^ We observed a significant loss of monomeric purity, an increase in molar mass (Figure 5), an increase in net hydrophobicity (Figure S 5), accompanied with an increase in the presence of submicron and subvisible particles at exposure to 80°C (Figure 6b), and an increase in Z-average, measured by DLS (Figure 6d). This implies the presence of insoluble and irreversible large IgE aggregates after thermal stress. The trends observed with IgE correlate with previous findings, which showed that an anti-streptavidin IgG1 increased matrix retention time and molar mass (g/mol) in response to 0–20 min at 67°C, measured by AF4.^61^ AF4 has proven effective in preserving native aggregation states of therapeutic peptides,^62^ implying the technique is nondestructive and capable of preserving irreversible and covalent aggregate states for more accurate representation of thermal stress consequences. Previous work has suggested that IgE may be more heat-labile than other antibody isotypes, incubation of IgE at 56°C significantly reduces anaphylactic activity, whereas significant IgG denaturation become notable at 65°C.^25^ At neutral pH, the unfolding temperature of certain IgE Fc regions occurs at 15°C lower than IgG molecules (T_M_ 55°C for Fcϵ Cϵ3 Cϵ4 and T_M_ 70°C for Fcγ Cγ3).^25,63^ This could have relevance in the context of IgE formulation development, as resistance to temperature excursions (elevated temperature) is key to maintaining conformational stability and retention of therapeutic activity during development.^64^

While therapeutic antibodies are unlikely to encounter the selected thermal stress temperatures (56 °C and 80 °C) in real-world storage or transport, they served as accelerated stability indicators, to model antibody degradation pathways. Studies under extreme stress conditions may be useful in determining whether accidental exposures to temperature excursions are deleterious to the product.^65^ Studies have demonstrated that mean kinetic temperature modeling can be applied to evaluate temperature excursions during storage and transport, helping predict long-term stability trends.^66^ However, these long-term stability predictions, based on accelerated stability data, are generally unreliable for biopharmaceuticals, because degradation pathways are influenced by complex interactions between temperature, time, and concentration, which may not directly correlate with real-time storage conditions.^30^ Future work must explore whether high-temperature degradation pathways differ from those occurring at lower temperatures (5 °C, 25 °C, and 40 °C), and any direct extrapolation must be carefully validated.^30^

Exposure to FT stress did not induce significant aggregation as there was a high degree of monomeric purity (90.55%) detected in samples relative to control, minimal changes in molar mass (Figure 5c,d), no detectable shifts in net hydrophobicity (Figure S5), and negligible submicron and subvisible particulate formation (Figure 6). While FT stress during transportation and storage is known to result in protein phase separation, salt crystallization, ice crystal formation, and abnormal refolding to induce aggregation,^67,68^ IgG has been shown under optimal formulation conditions to maintain physical stability after a single FT,^69^ but not after multiple cycles.^70^

It is important to evaluate the effect of agitation stress on IgE aggregation, as it is common for mAbs to encounter this during production (cell culture, purification, formulation, product filling) and shipping.^12^ Physical instability arising from agitation stress is induced by shear or interfacial effects in which the protein adsorbs to the air–water interface, leading to unfolding, hydrophobic core exposure, and aggregation that leads to cavitation, local thermal effects, and bubble entrapment.^71^ Our findings show that samples exposed to agitation stress retained a high monomeric purity (87.4%) and a molar mass distribution comparable to the unstressed samples (Figures 7 and 8a). Conversely, NTA measured the presence of a significant concentration of submicron and subvisible particulates (Figure 8a) and DLS validated this by showing formation of a new peak at 920.1 nm (Figure 8c). These observations suggest that agitation stress induces soluble, reversible, and low molecular mass aggregation, which were diluted by the running buffer during AF4 and SEC analyses. This correlates with previous research where the dilution of IgG2 (agitated for 24 hours) in acetate formulation buffer (pH 5.5) caused a reduction in measured particulate concentration by a high accuracy (HIAC) liquid particle counting method, in comparison to agitated samples that were not diluted in buffer.^72^ On the other hand, our DLS and NTA data correlate with previous findings which show that the hydrodynamic radius, total subvisible particle concentration (per mL), and total high-molecular weight species (HMWS) (%) of various mAb formats (100 mg/mL) increased after 24 hours of vertical 800 rpm agitation stress compared to the untreated control.^73^ Our work is limited by use of 2 mm headspace, or ~95% container fill volume. Similar studies on the impact of air–water interface during mechanical agitation use 70% fill volume.^74^ Any commercial development of MOv18 must come with further agitation studies using different parameters.

Early discovery findings on antibody aggregation play a crucial role in informing future commercial development. Our findings may help identify developability risks,^75^ optimize manufacturability,^76^ and refine engineering strategies to enhance stability.^77^ For example, a study on bispecific antibody manufacturability assessment highlighted how early-stage evaluations can identify and mitigate agitation-induced aggregation risks and streamline commercial production.^78^ Initial aggregation characterization can also connect to long-term stability predictions through advanced modeling and kinetic analysis. A study on the “Aggregation Time Machine” showed that short-term kinetic data can be used to predict long-term antibody stability, accelerating formulation optimization.^79^ Similarly, a study modeling an accelerated stability assessment of infliximab and biosimilars employed SEC and liquid chromatography techniques to assess stability over extended periods.^80^

Our findings also have implications for implementing packaging design and formulation strategies during transportation that mitigate for agitation risks, suggesting that exposure to agitation should be minimized, though agitation-induced aggregation is generally reversible. A small amount of soluble aggregates (5–10%) is acceptable in biologics due to the impracticality of removing them.^81^ Moreover, soluble aggregates are more favorable than insoluble aggregates, as they may reverse upon dilution following intravenous administration. With the AF4-UV and SEC-UV approaches that use carrier liquids and mobile phases, respectively, we saw the loss of some aggregates confirming the presence of reversible soluble agglomerates in our samples. These aggregates were present when analyzed by DLS and NTA sizing techniques.

Furthermore, the variability observed in the size distribution data obtained from the AF4-MALS-DLS method can be attributed to inherent limitations of light scattering techniques. As presented in Table S 3, the size values measured by MALS-DLS showed considerable variation. This inconsistency is likely due to poor scattering from individual particles, combined with a fundamental limitation of DLS – it assumes particle sphericity.^82^ Analysis of the AF4-MALS fractograms revealed that the signal intensity of the samples was too low to accurately determine the size, as both the radius of gyration (R_g_) and hydrodynamic radius (R_h_) were derived from angular scattering intensity, while the mass was calculated from total scattering intensity.^83,84^ In future studies, analysis of sample concentration may help improve signal strength and reduce variability in light scattering-based analyses. Conformational stability could also be further explored with nanoDSF/DSC experiments to determine unfolding/aggregation temperatures, which were deemed beyond the scope of this initial screening assay cascade

Overall, our results suggest that the IgE prototype studied demonstrates optimal physical stability at pH 6.5, which is resistant to FT-induced aggregation but not to thermal stress at elevated temperatures, or agitation stress. Current approaches to the analysis of IgG formulation stability are limited to SEC, and SDS-PAGE, whereas in this study we also demonstrated that AF4 is effective in separating and quantifying different size and molecular weight subspecies. Beyond formulation interventions, sequence engineering strategies such as the modulation of solvent accessible electrostatic and hydrophobic patches, and isoelectric manipulation^85^ may offer the scope to design stable IgE molecules.^86^ Additional studies are required to ascertain the impact of IgE aggregation on immunogenicity and their therapeutic efficacy in potency assays.

## Conclusions

The goal of this work was to provide a more detailed understanding of the physical stability of the first therapeutic IgE to be manufactured at scale, under different formulation pH conditions, and in response to environmental stressors encountered during an mAb product lifecycle. We implemented a range of bioanalytical techniques to study changes occurring in the physical stability of IgE. Overall, we show resistance to a loss of monomeric purity in response to formulation and the stress conditions tested, with minimal emergence of submicron particulates in the presence of FT cycling and lower temperature thermal stresses.

To our knowledge, this is the first report of an IgE formulation stability. Our findings can guide the development of future formulations of IgE-based immunotherapies in the field of AllergoOncology.

## Supplementary Material

KMAB-2025-0090.R3-Final SuppInformation.docx

## Data Availability

All data created during this research are openly available from the University of Strathclyde-Pure, at https://doi.org/10.15129/1f1c7b31-8be2-4434-b786-f21d9bf8f236.
