# Supplementary material for: Assessment of biophysical properties of the first-in-class anti-cancer IgE antibody drug MOv18 IgE demonstrates monomeric purity and stability
Source: MAbs. 2025 May 28;17(1):2512211. doi: 10.1080/19420862.2025.2512211 (PMC12123954; doi:10.1080/19420862.2025.2512211)
Supplement: KMAB-2025-0090.R3-Final SuppInformation.docx [file KMAB_A_2512211_SM8494.docx]

**Supplementary Information**

**Assessment of biophysical properties of the first-in-class anti-cancer IgE antibody drug MOv18 IgE demonstrates monomeric purity and stability**

Paul Considine*^1^, Panida Punnabhum*^1^, Callum G. Davidson^1^, Georgina B. Armstrong^1,2^, Michaela Kreiner^1,3^, Heather J Bax^4^, Jitesh Chauhan^4^, James Spicer^5,6^, Debra H Josephs^4,5,6^, Sophia N Karagiannis^4,7$^, Gavin Halbert^1,3$^, and Zahra Rattray^1$^

1. Strathclyde Institute of Pharmacy and Biomedical Sciences, University of Strathclyde, Glasgow, UK.
2. Drug Substance Development, GlaxoSmithKline, Gunnels Wood Road, Stevenage, UK
3. Cancer Research UK Formulation Unit, Strathclyde Institute of Pharmacy and Biomedical Sciences, University of Strathclyde, Glasgow, UK.
4. St. John's Institute of Dermatology, School of Basic and Medical Biosciences & KHP Centre for Translational Medicine, Guy’s Hospital, King's College London, London, United Kingdom
5. School of Cancer and Pharmaceutical Sciences, King’s College London, London, United Kingdom
6. Cancer Centre, Guy’s and St Thomas’ NHS Foundation Trust, London, United Kingdom
7. Breast Cancer Now Research Unit, School of Cancer & Pharmaceutical Sciences, King's College London, Innovation Hub, Guy's Cancer Centre, London, United Kingdom

*Both authors contributed to this work equally.

$corresponding author(s): [gavin.halbert@strath.ac.uk](mailto:gavin.halbert@strath.ac.uk); [zahra.rattray@strath.ac.uk](mailto:zahra.rattray@strath.ac.uk); [sophia.karagiannis@kcl.ac.uk](mailto:sophia.karagiannis@kcl.ac.uk)

**
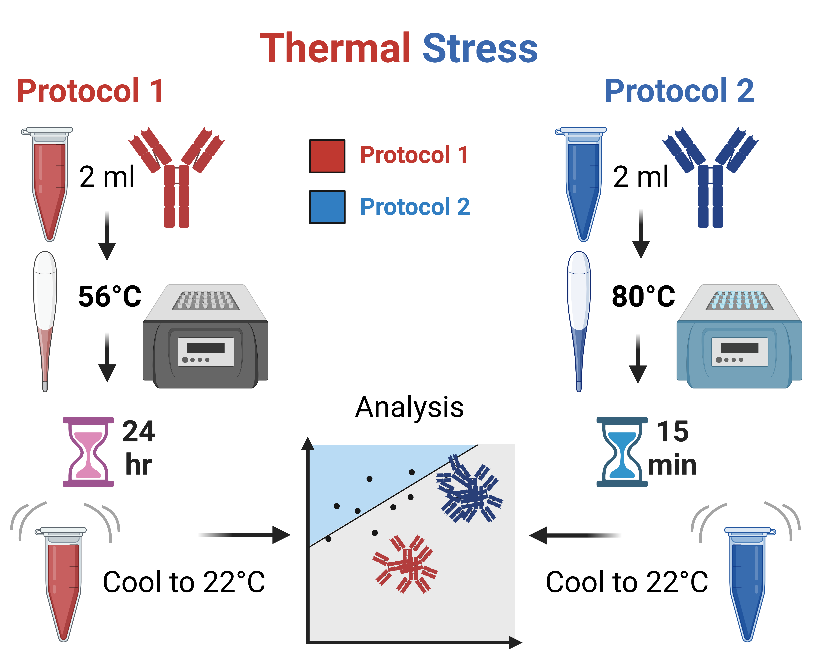
**

Figure S 1 Illustration of thermal stress protocol 1 & 2. (Created with Biorender.com).

**A**

**
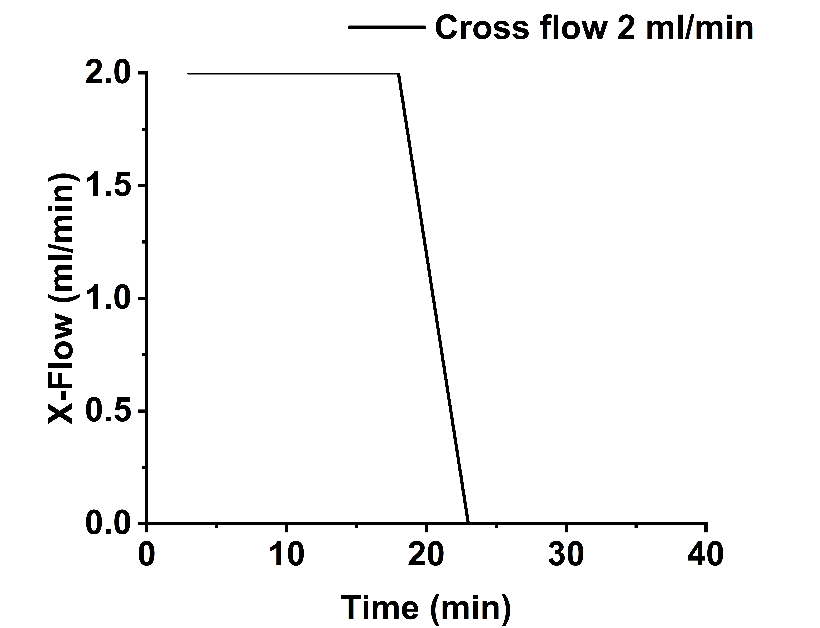
**

**B**

| Method | Delay time  (min) | Detector flow (mL/min) | Focus Step | | | Elution step | | | | Rinse step | |
| --- | --- | --- | --- | --- | --- | --- | --- | --- | --- | --- | --- |
|  |  |  | Injection min | Injection Flow (mL/min) | Cross flow (mL/min) | Duration  (min) | Cross flow (mL/min) | Mode | Exponent | Tip flow (mL/min) | Transition time (min) |
| 4 | 4 | 0.5 | 2 | 0.2 | 2 | 15 | 2 | Constant | 0 | 0.5 | 5 |
|  |  |  |  |  |  | 5 | 2 | Linear | 1 |  |  |
|  |  |  |  |  |  | 10 | 0 | Constant | 0 |  |  |

Figure S 2 Final AF4 Method Profile at 2XF (ml/min (A) and flow parameters and conditions (B).

*
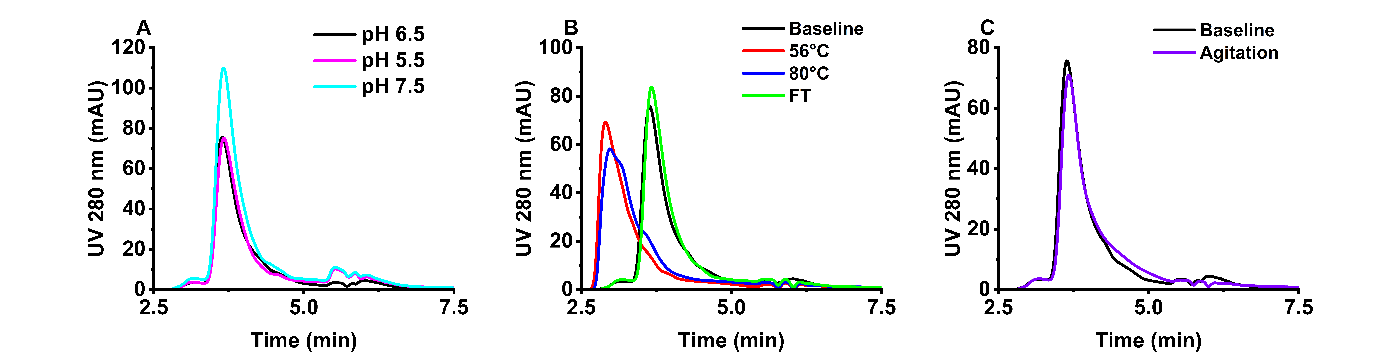
*

**Figure S 3 Analysis of size distribution by size exclusion chromatography (SEC).** IgE at 1 mg/ml was analysed from (A) baseline untreated at pH 6.5, after buffer exchanged into pH 5.5 and pH 7.5, (B) exposure to 56°C for 24 hours, 80°C for 15 mins, five freeze-thaw cycles (FT), or (C) 48 hour shaking agitation stress, (n=3).

*
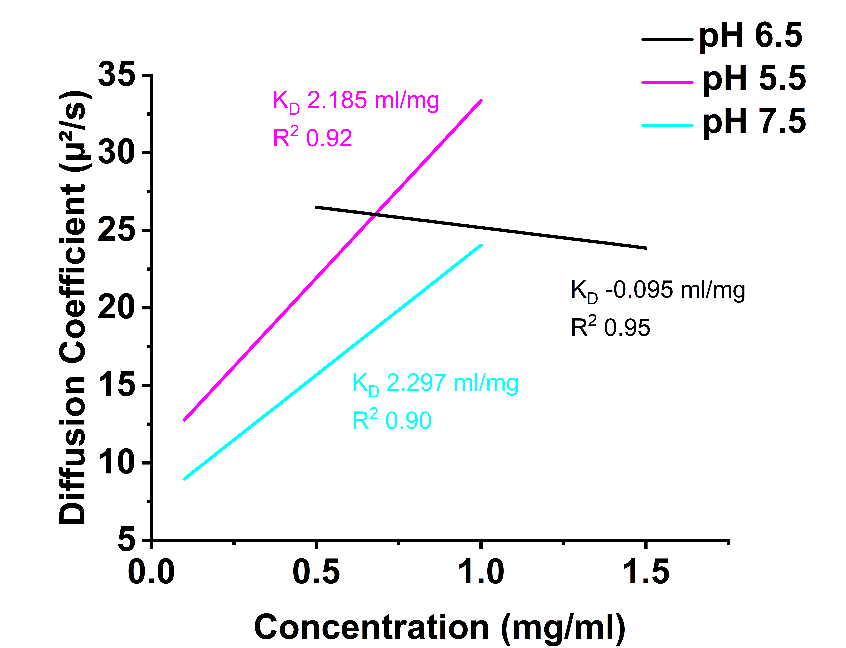
*

Figure S 4 The DLS interaction parameter was inconclusive. Diffusion coefficient vs. concentration 0.1 mg/ml to 1.5 mg/ml, K_D_ calculation, and R^2^ values, (n=3). The K_D_ was calculated by using linear regression analysis (slope/ intercept).


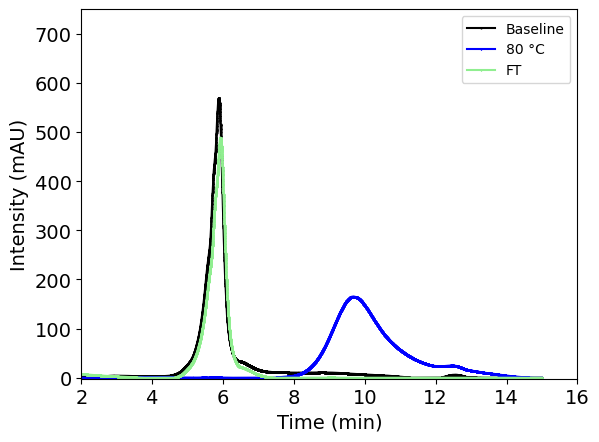


Figure S 5 Net hydrophobicity assessment of thermally stressed samples. Data generated from analytical Hydrophobic Interaction Chromatography (HIC). 10 μg of IgE samples, diluted to 1 mg/mL in PBS (80°C for 15 mins, five freeze-thaw cycles (FT)) were injected onto a butyl column to assess shifts in retention time and peak broadening in accordance with net hydrophobicity and polydiversity of hydrophobic species, (n=2).

Table S 1 AF4 Elution Time Range. Data from asymmetric-flow-field flow fractionation (AF4). Details of the time range (min) used to identify each size species (fragment, monomer, dimer, oligomer, aggregate) using AF2000 software. IgE control (Baseline/ pH 6.5), thermal stress 56°C, thermal stress 80°C, freeze thaw (FT) stress, or buffer exchange to pH 7.5 or pH 5.5. (n=3).

|  | **Retention time (min)** | | | | |
| --- | --- | --- | --- | --- | --- |
|  | **Fragment** | **Monomer** | **Dimer** | **Oligomer** | **Aggregate** |
| **pH 5.5** | 6.8 – 9.1 | 9.6 – 14.5 | 14.9 – 17.6 | n.d. | 22.7 – 33.5 |
| **Baseline (pH 6.5)** | 6.7 – 8.3 | 8.8 – 12.8 | 13.1 – 15.9 | n.d. | 21.6-28.8 |
| **pH 7.5** | 6.9 – 8.8 | 8.9 – 13.2 | 13.3 – 16 | n.d. | 22.1 – 28.8 |
| **56°C** | 6.2 – 9 | 9.1 – 11.5 | 11.5 – 13.9 | 13.9 – 18.1 | 18.1 – 31.8 |
| **80°C** | 7.3 – 8.1 | 8.1 – 10.1 | 10.1 – 12.3 | 12.4 – 18.5 | 18.6 - 32 |
| **FT** | 6.2 – 8.4 | 8.5 – 12.1 | 12.4 – 15.3 | n.d. | 21.7 - 28 |
| **Agitation** | 6.6 – 8.3 | 8.3 – 12.7 | 13 – 15.5 | n.d. | 21.7 – 28.3 |

Table S 2 SEC Mean retention time of peaks. Data from analytical size exclusion chromatography. The retention time (min) of the highest point of each peak separated in SEC, used to identify each size species (fragment, monomer, oligomer/aggregate) using software ROI markers. IgE control (Baseline/ pH 6.5), thermal stress 56°C, thermal Stress 80°C, freeze thaw (FT) stress, or buffer exchange to pH 7.5 or pH 5.5. (n=3) ± S.D. N.d. – not detected. There was not sufficient content within the monomer range (3.6-3.7 min) for samples 56°C & 80°C.

|  | **Mean Retention time of Peak ± S.D. (min)** | | |
| --- | --- | --- | --- |
|  | **Fragment** | **Monomer** | **Oligomer/ Aggregate** |
| **pH 5.5** | 5.25 (± 0.56) | 3.67 (± 0.002) | 3.15 (± 0.005) |
| **Baseline (pH 6.5)** | 5.8 (± 0.19) | 3.64 (± 0.03) | 3.17 (± 0.01) |
| **pH 7.5** | 5.38 (± 0.59) | 3.66 (± 0.003) | 3.17 (± 0.02) |
| **56°C** | 5.76 (± 1.08) | n.d. | 2.91 (± 0.02) |
| **80°C** | 5.891 (± 0.21) | n.d. | 2.98 (± 0.01) |
| **FT** | 5.55 (± 0.54) | 3.67 (± 0.002) | 3.2 (± 0.04) |
| **Agitation** | 5.86 (± 0.005) | 3.67 (± 0.002) | 3.18 (± 0.003) |

Table S 4 Thermal stress and pH changes caused fluctuations in particle concentration and size. Data determined by nanoparticle tracking analysis (NTA). (a) Mean particle concentration (#/ ml), mean particle size, mode, and percentile values (D10, D50, D90) (nm) of IgE. IgE control (Baseline), thermal stress 56°C, thermal Stress 80°C, freeze thaw stress, or buffer exchange to pH 7.5 or pH 5.5. (n=3) ± S.D.

| **Stress** | **Mean Particle Concentration (#/ ml) (± S.D.)** | **Mean Size (nm) (± S.D.)** | **Mode (nm) (± S.D.)** | **D10 (nm) (± S.D.)** | **D50 (nm) (± S.D.)** | **D90 (nm) (± S.D.)** |
| --- | --- | --- | --- | --- | --- | --- |
| **pH 6.5 (Baseline)** | 0 ± 0 | 0 ± 0 | 0 ± 0 | 0 ± 0 | 0 ± 0 | 0 ± 0 |
| **pH 5.5** | 1.02 x 10^8^ ± 3.18 x 10^7^ | 172.37 ± 1.65 | 160 ± 9.89 | 107.90 ± 7.75 | 168.80 ± 1.54 | 244.63 ± 23.43 |
| **pH 7.5** | 7.92 x 10^6^ ± 1.80 x 10^6^ | 218.43 ± 3.3 | 215.47 ± 4.72 | 144.87 ± 12.83 | 217.90 ± 5.8 | 294.40 ± 8.37 |
| **56 ℃** | 0 ± 0 | 0 ± 0 | 0 ± 0 | 0 ± 0 | 0 ± 0 | 0 ± 0 |
| **80 ℃** | 5.85 x 10^7^ ± 2.31 x 10^6^ | 137.9 ± 1.66 | 145.80 ± 9.40 | 82.07 ± 7.25 | 140.27 ± 1.02 | 186.10 ± 5.78 |
| **FT** | 3.83 x 10^5^ ± 4.71 x 10^5^ | 12.27 ± 4 | 12.17 ± 6.84 | 10.97 ± 6.15 | 12.23 ± 6.9 | 13.57 ± 7.79 |
| **Agitation** | 1.03 x 10^8^ ± 1.85 x 10^7^ | 160.53 ± 27.03 | 146.07 ± 6.86 | 63.07 ± 11.93 | 145.27 ± 24.23 | 231.63 ± 67.02 |

**Table S 3 Thermal stress and pH changes caused fluctuations in Radius of gyration, hydrodynamic radius, and shape factor.** Data determined by AF4-MALS-DLS. (nm) of IgE. R_g_ Radius of gyration, R_h_ Hydrodynamic radius, IgE control (Baseline), thermal stress 56°C, thermal Stress 80°C, freeze thaw stress, or buffer exchange to pH 7.5 or pH 5.5. (n=3) ± S.D. Statistical analysis was completed using a Tukey simultaneous test for difference of means. * = p<0.05, ** = p<0.01, *** = p<0.001, **** = p<0.0001, ns – not significant versus baseline. n/a - not applicable.

| **Stress** | **R_g_ (nm) (± S.D.)** | | | | **R_h_ (nm) (± S.D.)** | | | | **Shape factor (R_g_/R_h_)** | | | |
| --- | --- | --- | --- | --- | --- | --- | --- | --- | --- | --- | --- | --- |
|  | **Monomer** | **Dimer** | **Oligomer** | **Aggregate** | **Monomer** | **Dimer** | **Oligomer** | **Aggregate** | **Monomer** | **Dimer** | **Oligomer** | **Aggregate** |
| **pH 6.5 (Baseline)** | 7.3 ± 4.3 | n/a | 16.4 ± 9.5 | 28.7 ± 7.7 | 5.2 ± 1.1 | n/a | 5.7 ± 2.9 | 25 ± 20 | 1.4 ± 0.8 | n/a | 3 ± 2 | 2.1 ± 1.4 |
| **pH 5.5** | 9.6 ± 6.1 (ns) | n/a | 36.5 ± 7.2 (ns) | 61.0 ± 0.5 (**) | 5.0 ± 0.2 (ns) | n/a | 6.1 ± 0.9 (ns) | 17.4 ±2.3 (****) | 1.9 ± 1.1 (ns) | n/a | 6 ± 2 (ns) | 3.6 ± 0.4 (ns) |
| **pH 7.5** | 11.5 ± 4.1 (ns) | n/a | 33.9 ±14.4 (ns) | 51.5 ± 5.7 (*) | 8.5 ± 3.8 (ns) | n/a | 25.7 ± 23.9 (ns) | 75.6 ± 97.6 (ns) | 1.8 ± 1.0 (ns) | n/a | 2.7 ± 1.5 (ns) | 6 ± 4 (ns) |
| **56 ℃** | 15.8 ± 6.8 (ns) | 14.4 ± 9 | 12.9 ± 4.6 (ns) | 23.9 ± 2.4 (ns) | 5.3 ± 0.7 (ns) | 5 ± 1 | 6.9 ± 0.6 (ns) | 9.4 ± 0.2 (ns) | 3 ± 1 (ns) | 5 ± 1 | 1.9 ± 0.8 (ns) | 2.5 ± 0.2 (ns) |
| **80 ℃** | 16.2 ± 6.5 (ns) | 31.8 ± 21 | 13.8 ± 14.7 (ns) | 24.8 ± 2.9 (ns) | 6 ± 1 (ns) | 6.6 ± 0.4 | 8.9 ± 1 (ns) | 14.3 ± 7.4 (ns) | 3 ± 1 (ns) | 6.6 ± 0.4 | 1.7 ± 1.8 (ns) | 2.4 ± 1.3 (ns) |
| **FT** | 4.5 ± 2.8 (ns) | n/a | 20.4 ± 11.9 (ns) | 41.2 ± 9.5 (ns) | 5.4 ± 0.6 (ns) | n/a | 7 ± 4 (ns) | 12.1 ± 13 (ns) | 1 ± 0.5 (ns) | n/a | 4 ± 4 (ns) | 11.2 ± 8.3 (ns) |
| **Agitation** | 14.8 ± 0.8 (ns) | n/a | 11.2 ± 4.1 (ns) | 31 ± 13 (ns) | 5.3 ± 0.7 (ns) | n/a | 5.6 ± 2.3 (ns) | 13.3 ± 12.9 | 2.8 ± 0.2 (ns) | n/a | 2.5 ± 1.4 (ns) | 7.8 ± 8.4 (ns) |
